# FLOTAC and Mini-FLOTAC for uro-microscopic diagnosis of *Capillaria plica* (syn. *Pearsonema plica*) in dogs

**DOI:** 10.1186/1756-0500-7-591

**Published:** 2014-09-02

**Authors:** Maria Paola Maurelli, Laura Rinaldi, Giuseppe Rubino, Riccardo Lia, Vincenzo Musella, Giuseppe Cringoli

**Affiliations:** Department of Veterinary Medicine and Animal Productions, University of Naples Federico II CREMOPAR, Campania Region, Naples, Italy; Department of Animal Production, University of Bari “Aldo Moro”, Valenzano, BA Italy; Department of Veterinary Medicine, University of Bari “Aldo Moro”, Valenzano, BA Italy; Department of Health Science, University of Catanzaro Magna Graecia, Catanzaro, Italy

**Keywords:** *Capillaria plica*, Dog, FLOTAC, Mini-FLOTAC, Sedimentation technique

## Abstract

**Background:**

*Capillaria plica* (syn. *Pearsonema plica*) is a nematode that resides in the urinary bladder and rarely in ureters or in the kidney pelvis of various carnivores, especially foxes and dogs. Urine sedimentation technique is actually the only diagnostic tool that permits the identification of *C. plica* eggs, but its sensitivity is low and when an infection is suspected (or when it is necessary to confirm treatment efficacy) more than one examination of urine sediment should be performed. The present paper reports a clinical case of natural *C. plica* infection in a dog from southern Italy. In addition, two new techniques, FLOTAC and Mini-FLOTAC, were used for the diagnosis of *C. plica* in dog urine and compared with the technique of sedimentation.

**Results:**

Using FLOTAC with fresh urine and sodium chloride as flotation solution, were obtained the best results for the diagnosis of *C. plica* in dog urine in term of eggs counted (mean eggs per 10 ml of urine = 70.3 FLOTAC *vs* 40.3 Mini FLOTAC *vs* 32.8 sedimentation) and coefficient of variation (CV%) (6.2 FLOTAC *vs* 13.4 Mini-FLOTAC *vs* 32.9 sedimentation).

**Conclusions:**

The FLOTAC was the more sensitive method, but also the Mini-FLOTAC could be a valid alternative diagnostic method because gave better results than the classical sedimentation and can be used in place of the FLOTAC in laboratories where the centrifugation step cannot be performed.

## Background

*Capillaria plica* (Syn. *Pearsonema plica*) [1], commonly known as the “bladderworm”, is a nematode that resides in the urinary bladder and rarely in ureters and kidney pelvis of various carnivores, especially foxes and dogs [2–4]. *C. plica* has an indirect life cycle and involves an earthworm as the intermediate host. Once the definitive host eats earthworms with the first stage larvae, these moult to the second stage and then to the third stage within the wall of the small intestine. Finally, the larvae reach the bladder, where they moult to adults and embed themselves deep into bladder mucosa [5]. The prepatent period in dogs has been reported to be around 8–10 weeks [6]. *C. plica* is considered to be of low pathogenic significance and in most cases, the parasite establish only asymptomatic infections [7]. The symptomatic cases described in domestic dogs have showed dysuria, haematuria, pollakiuria, polydipsia and urinary incontinence [8–10]. Recently, *C. plica* has been also suggested to be a contributing factor in glomerular amyloidosis in dogs [5].

Urine sedimentation technique is actually the only diagnostic tool that permits the identification of *C. plica* eggs [5]. It is a qualitative method and its sensitivity is low and when an infection is suspected (or when it is necessary to confirm treatment efficacy) more than one examination of urine sediment should be performed [11].

The FLOTAC techniques are quantitativce methods described for the copromicroscopic diagnosis of parasites in humans and animals [12] and permit a multivalent detection of dog parasites including *Crenosoma vulpis* [13], *Spirocerca lupi* [14], *Angiostrongylus vasorum* [15] and *Ancylostoma caninum* [16]. Interestingly, FLOTAC has been also successfully utilized for the diagnosis of *Schistosoma haematobium* in human urine [17].

Mini-FLOTAC is an evolution of FLOTAC conceived to perform multivalent faecal egg counts through an easy approach. It has a simple design, which is based on only two components, the base and the reading disc. The device includes also two 1-ml flotation chambers designed for optimal examination of faecal sample suspensions (total volume = 2 ml) [18]).

Mini-FLOTAC has been already validated for the diagnosis of most important human nematodes (e.g. soil-transmitted helminths) and trematodes (e.g. *Schistosoma*) [19] and showed promising results in veterinary parasitology for the diagnosis of helminths (e.g. ascarids, hookworms, trichurids, gastro-intestinal nematodes and liver flukes) in pets and livestock [20].

The aims of this work are: 1) to evaluate two new quantitative techniques, FLOTAC and Mini-FLOTAC, for the diagnosis of *C. plica* in dog urine; 2) to compare these two innovative techniques with the technique of sedimentation, in terms of sensitivity and precision.

## Results

**Figure 1:**
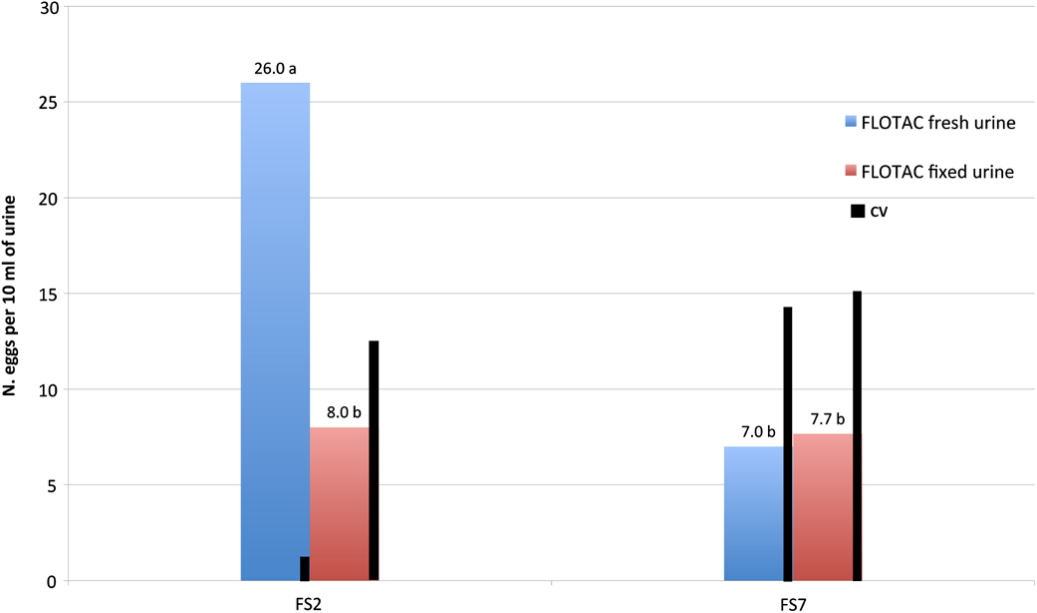
**Mean** ***C. plica*** **eggs and coefficient of variation (CV%) detected by FLOTAC using two flotation solutions (FS2 and FS7) on fresh and fixed urine.** Significant differences (P < 0.05) for different letters (a, b).

The Figure 1 shows the mean *C. plica* eggs per 10 ml of dog urine calculated for each of two flotation solutions (FSs): sodium chloride (FS2) and zinc sulphate (FS7) and for two preservation methods: fresh and fixed urine, along with the coefficient of variation (CV), using FLOTAC.

*C. plica* eggs were detected with both FSs either using fresh urine and fixed urine. However, the highest count (26.0 eggs per 10 ml) and lowest CV (3.8%) were obtained with fresh urine (P = 0.04) and using FS2 (P =0.04) i.e., sodium chloride.

**Figure 2:**
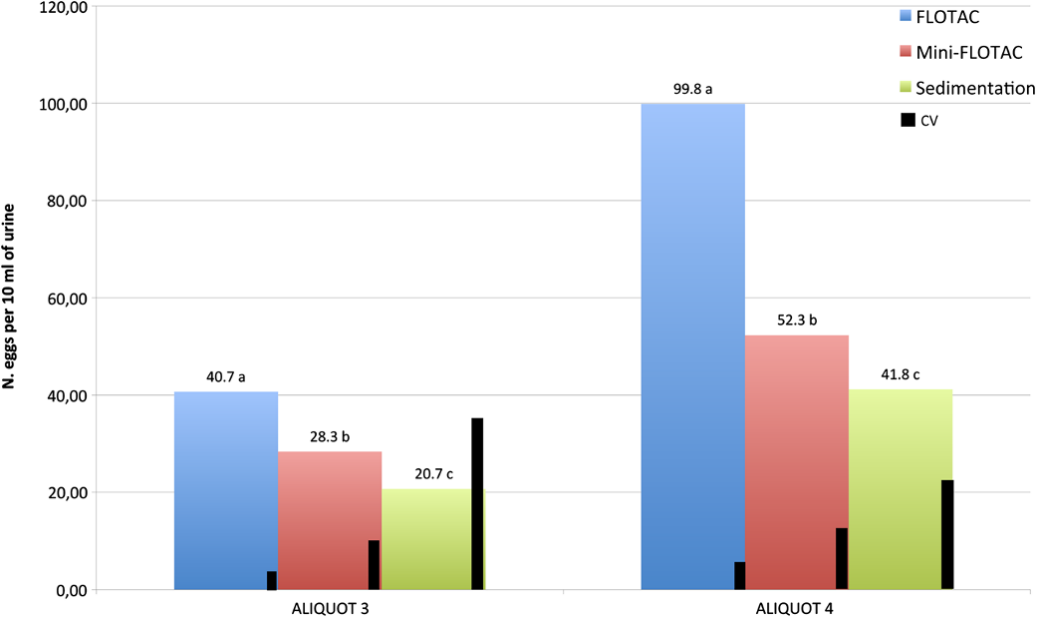
**Mean** ***C. plica*** **eggs and coefficient of variation (CV%) detected by FLOTAC, Mini-FLOTAC and urine sedimentation techniques.** Two aliquots (aliquot 3 and 4) of fresh urine were used. FS2 solution was used for FLOTAC and Mini-FLOTAC. Significant differences (P < 0.05) for different letters (a, b, c).

All the three techniques (FLOTAC, Mini-FLOTAC and urine sedimentation) were capable to detect *C. plica* eggs. For aliquot 3, the mean number of *C. plica* detected with FLOTAC was significantly higher than those detected by Mini-FLOTAC and sedimentation (40.7 eggs per 10 ml of urine *vs* 28.3 and 20.7, respectively); in addition the CV% detected with FLOTAC was lower than the values detected by the other techniques (5.5 *vs* 12.8 and 36.0, respectively). Also for the aliquot 4, FLOTAC gave higher mean of *C. plica* detected (99.8 eggs per 10 ml of urine *vs* 52.3 and 44.8, respectively) and lower CV% (6.8 *vs* 14.0 and 29.7 respectively) than the other two techniques (Figure 2).

## Discussion

Reports of *C. plica* infection in dogs and cats are limited and the actual prevalence could be underestimated. In Italy only one clinical case in a hunting jagd terrier in Parma [5] and one in an European cat from Pisa [21] have been reported so far. Many studies, however, have revealed a high prevalence in red foxes in several European countries, with prevalence ranging from 50% to 80% [22]. Thus, fox populations are likely the main source of infection for hunting dogs [5].

The dog examined in our present study was born in Spain where *C. plica* is widespread in foxes [23] and lived in the Apulia Region for less of one year. None of other dogs that lived together presented the same symptomatology.

The treatment with febendazole at a dose rate of 50 mg/kg/day for 10 days was successfully used, as reported also in other cases in literature [6, 21, 24].

## Conclusions

The results of this work provide important new information on the performance of available methods for the *intra vitam* diagnosis of *C. plica* in dogs. The findings have suggested that the FLOTAC, using fresh urine and sodium chloride (FS2), may improve the ability to accurately diagnose capillariosis in dog urine and permit to overcome the limits of the “classical” sedimentation method. The FLOTAC has yet used successfully also for the diagnosis of *S.haematobium* in human [17], and so could be proposed as good method for diagnosis of parasites in urine. An alternative diagnostic method is Mini-FLOTAC that gave better results than the classical urine sedimentation technique and can be used in place of the FLOTAC in laboratories where the centrifugation step cannot be performed. However, further studies are required to confirm the findings of the present case report.

## Methods

### Case report

A 4-year-old Labrador Retriever, male, born in Spain and living in the Apulia Region (southern Italy) was presented to the referring clinician with a recurrence of dermatitis at flat thighs, abdomen and testicles. The dog was treated previously with cortisone and fluid therapy with no positive outcomes. Clinical diagnosis showed palpable abdomen, internal organs evaluable and a productive cough. The dog was regularly vaccinated and treated against ectoparasites.

Chest x-ray examination revealed not altered structures. Abdominal ultrasound revealed chronic cystitis with thickening of the bladder wall with irregular margins. A serum chemical profile and complete blood count (CBC) revealed eosinophilia, whereas other parameters were within normal limits.

A macroscopic examination of urine revealed haematuria, while a microscopic examination of urinary sediment revealed the presence of several *C. plica* eggs. The dog was treated with febendazole at a dose rate of 50 mg/kg/day for 10 days and showed a rapid recovery, with resolution of all clinical signs.

### Flotation solutions choice for FLOTAC and Mini-FLOTAC and urine preservation

To determine the optimum flotation solution for FLOTAC and Mini-FLOTAC and the possibility to preserve urine, two FSs: sodium chloride (FS2), specific gravity (s.g. 1200) and zinc sulphate (FS7) (s.g. 1350) were compared using either fresh urine and urine fixed with 5% formalin (because could be a good alternative method of preservation of urine from collection until analysis).

For this aim one hundred and twenty ml of urine were collected from the *C. plica* infected dog (see *Case report* paragraph).

The urine was divided in two aliquots (Aliquot 1 and Aliquot 2) of 60 ml each: Aliquot 1 was analyzed as fresh, Aliquot 2 was fixed with 3 ml of a 37% formaldehyde solution and analyzed after 7 days. Each of two aliquots was carefully homogenized and 6 tubes were filled with 10 ml of urine, to have 3 replicates for each of the two FSs used.

The tubes were centrifuged at 240 g for 5 minutes and the supernatant poured off and discarded. The tube with the remaining pellet was then filled with each FS until 11 ml level mark and slowly agitated.

The suspension was transferred into the two chambers of a FLOTAC apparatus using a disposable pipette. The apparatus was centrifuged at 120 g for 5 min, and translated to separate the floating eggs from the urinary debris. All eggs visible through the ruled window of the reading disc were counted under a microscope at 100× total magnification.

### Comparison of FLOTAC, Mini-FLOTAC and urine sedimentation techniques

The following three techniques were compared for the diagnosis of *C. plica* in dog urine: the FLOTAC basic technique [12, 17], the Mini-FLOTAC technique [18] and the urine sedimentation technique [25].

Fresh urine and FS2 (for FLOTAC and Mini-FLOTAC) were used based on the results obtained from the previous experiment (see *Flotation solutions choice for FLOTAC and Mini-FLOTAC and urine preservation* paragraph).

Two aliquots (Aliquot 3 and Aliquot 4) of 200 ml each of urine were collected from the same dog naturally infected by *C. plica*. Each aliquot of urine was accurately homogenized and divided in 18 tubes each filled with 10 ml of urine, to have 6 replicates for each of the three diagnostic method. The tubes were randomly assigned to the three techniques.

For the urine sedimentation technique, the tubes were left to room temperature for 1 h, the supernatant was poured off [25] and the tubes were centrifuged at 2000 g for 2 minutes. After the supernatant was discarded, the pellet has been examined on a glass slide.

For the FLOTAC and Mini-FLOTAC techniques, the tubes were centrifuged at 240 g for 5 minutes, the supernatant was poured off and 6 ml of FS2 were added to tubes to fill the FLOTAC chambers, whereas 2 ml of FS2 were added to tubes to fill the Mini-FLOTAC chambers (for both techniques the entire pellet was examined).

For each diagnostic technique, *C. plica* eggs were counted for all replicates using a light microscope at 10× total magnification. The analytic sensitivity of all the three techniques was 1 egg per 10 ml of urine.

### Statistical analysis

The arithmetic mean eggs per 10 ml of urine, standard deviation (SD), and coefficient of variation (CV) were calculated for the two FSs, preservation method and diagnostic technique. Differences between FSs were analyzed using one-way ANOVA with post hoc Fisher’s least significant difference (LSD). All statistical analyses were carried out using STATA version 10.0 (Stata Corp.; Texas, USA). In addition, a likelihood ratio test of the equality of the CV of normally distributed populations was performed using software developed by the Statistical Services at the Forest Products Laboratory (USA; http://www1.fpl.fs.fed.us/covtestk.html).

### Consent

Written informed consent was obtained from the owner of dog to publish data and information in this report.
